# Acid-induced off-response of PKD2L1 channel in *Xenopus* oocytes and its regulation by Ca^2+^

**DOI:** 10.1038/srep15752

**Published:** 2015-10-27

**Authors:** Shaimaa Hussein, Wang Zheng, Chris Dyte, Qian Wang, JungWoo Yang, Fan Zhang, Jingfeng Tang, Ying Cao, Xing-Zhen Chen

**Affiliations:** 1 Membrane Protein Disease Research Group, Department of Physiology, Faculty of Medicine and Dentistry, University of Alberta, Edmonton, AB, Canada; 2 School of Life Sciences and Technology, Tongji University, Shanghai, China; 3 Membrane Protein Disease and Cancer Research Center, Hubei University of Technology, Wuhan, China

## Abstract

Polycystic kidney disease (PKD) protein 2 Like 1 (PKD2L1), also called transient receptor potential polycystin-3 (TRPP3), regulates Ca^2+^-dependent hedgehog signalling in primary cilia, intestinal development and sour tasting but with an unclear mechanism. PKD2L1 is a Ca^2+^-permeable cation channel that is activated by extracellular Ca^2+^ (on-response) in *Xenopus* oocytes. PKD2L1 co-expressed with PKD protein 1 Like 3 (PKD1L3) exhibits extracellular acid-induced activation (off-response, i.e., activation following acid removal) but whether PKD1L3 participates in acid sensing remains unclear. Here we used the two-microelectrode voltage-clamp, site directed mutagenesis, Western blotting, reverse transcriptase-polymerase chain reaction (RT-PCR) and immunofluorescence, and showed that PKD2L1 expressed in oocytes exhibits sustained off-response currents in the absence of PKD1L3. PKD1L3 co-expression augmented the PKD2L1 plasma membrane localization but did not alter the observed properties of the off-response. PKD2L1 off-response was inhibited by an increase in intracellular Ca^2+^. We also identified two intra-membrane residues aspartic acid 349 (D349) and glutamic acid 356 (E356) in the third transmembrane domain that are critical for PKD2L1 channel function. Our study suggests that PKD2L1 may itself sense acids and defines off-response properties in the absence of PKD1L3.

Polycystic kidney disease (PKD) protein 2 Like 1 (PKD2L1) belongs to the transient receptor potential (TRP) superfamily of which members are implicated in a number of sensory functions, such as detection of light, force, osmolarity, temperature, odor, taste and pain^1^,^2^. Human PKD2L1 gene is a homologue of PKD protein 2 (PKD2) and was first identified in 1998^3^. While mutations in PKD protein 1 (PKD1) and PKD2 account for about 85% and 15% of the cases in autosomal dominant PKD (ADPKD)^4^, respectively, PKD2L1 does not seem to be involved in the disease. In 1999, Chen *et al.* discovered the Ca^2+^-regulated non-selective cation permeability of PKD2L1^5^. We previously reported that human PKD2L1 expressed alone in *Xenopus* oocytes targets to the plasma membrane (PM) and displays the regulation of Ca^2+^-induced channel activation (on-response) by a number of factors including Ca^2+^, voltage, pH, amiloride analogs, large monovalent organic cations, troponin I, α-actinin, and receptor for activated protein kinase 1 (RACK1)^5^,^6^,^7^,^8^,^9^,^10^,^11^,^12^. In mammalian cells, outward rectifying cation permeability of PKD2L1, with or without co-expression of PKD protein 1 Like 1 (PKD1L1, a homologue of PKD1), was recently reported^13^. This group of researchers found that PKD2L1, present in primary cilia, regulates the ciliary Ca^2+^ concentration and sonic hedgehog signalling. Lack of PKD2L1 in mice was associated with defective hedgehog signalling and development of inverted colon^13^,^14^.

In mammalian cells and oocytes, PKD2L1, in complex with PKD protein 1 Like 3 (PKD1L3, another PKD1 homologue), was activated by acids in an off-response manner, i.e., the PKD2L1/PKD1L3 channel complex mediated a large current only after the removal of the extracellular acid^15^,^16^. These and subsequent studies^17^,^18^ indicated that PKD2L1 or PKD1L3 forms a novel extracellular acid sensor. Forming a candidate for sour taste sensation, PKD2L1 was shown to interact with PKD1L3^18^ in murine taste receptor type III cells. PKD2L1 and PKD1L3 were co-expressed around the pore region of taste buds in mouse circumvallate and foliate papillae^19^, but only PKD2L1 was found in other areas, including the fungiform and palate taste buds^15^,^18^. The involvement of the PKD2L1/PKD1L3 complex in sour tasting remains controversial as studies using knockout mice showed that PKD2L1, but not PKD1L3, is involved in sour tasting and acid sensing^17^,^20^,^21^. Further, these taste buds, regardless of whether expressing PKD1L3 or not, still have similar PKD2L1 distribution patterns with a specific localization to the pore region^18^,^21^. The PM localization of PKD2L1 alone was reported in both HEK cells and *Xenopus* oocytes by immunofluorescence, surface biotinylation and/or electrophysiology^5^,^12^,^13^,^16^,^22^. Co-expression of PKD1L3 substantially increased the PKD2L1 PM targeting, which presumably allowed the channel function to be observed more easily^15^,^16^,^23^,^24^.

PKD2L1/PKD1L3 channel complex responded to both weak and strong acids. It was reported that weak acids, such as citric acid, induce larger off-response currents in HEK cells expressing the PKD2L1/PKD1L3 complex than strong acids, such as HCl, at the same pH. All these acids possessed similar activation threshold values of around pH 3.0^18^. Controversially, it was reported later that the induced off-response for PKD2L1/PKD1L3 by strong (HCl and sulphuric) and weak (citric, malic, phosphoric, succinic and tartaric) acids are not significantly different in HEK cells^15^. In intact biological systems, PKD2L1-expressing cells were associated with activation thresholds of higher pH values. For example, the threshold value for taste receptor cells (TRCs) was found to be around pH 5.0^20^,^25^. Murine cerebrospinal fluid contacting neurons (CSF-cN), expressing endogenous PKD2L1, were very sensitive to changes in extracellular pH, in the pH range of 6.5–7.4^20^. Moreover, two negatively charged residues in the PKD2L1 pore loop, D523^26^ and D525^16^, have been identified to affect Ca^2+^ permeation and cation selectivity of the PKD2L1/PKD1L3 channel complex, and may thus be involved in channel gating and selectivity.

Despite recent significant progresses towards understanding the biological and physiological roles of PKD2L1 and PKD1L3, how PKD2L1 and/or PKD1L3 sense acid is not fully understood. In the current study, we employed *Xenopus* oocyte expression model to examine how acids and PKD1L3 modulate PKD2L1 channel activity. In addition, we examined roles of Ca^2+^ and intra-membrane acidic residues in this modulation. For this we utilized two-microelectrode voltage clamp in combination with mutagenesis, Western blotting, RT-PCR and immunofluorescence.

## Results

### Acid-induced off-response currents in oocytes expressing PKD2L1 with or without PKD1L3

**Figure 1:**
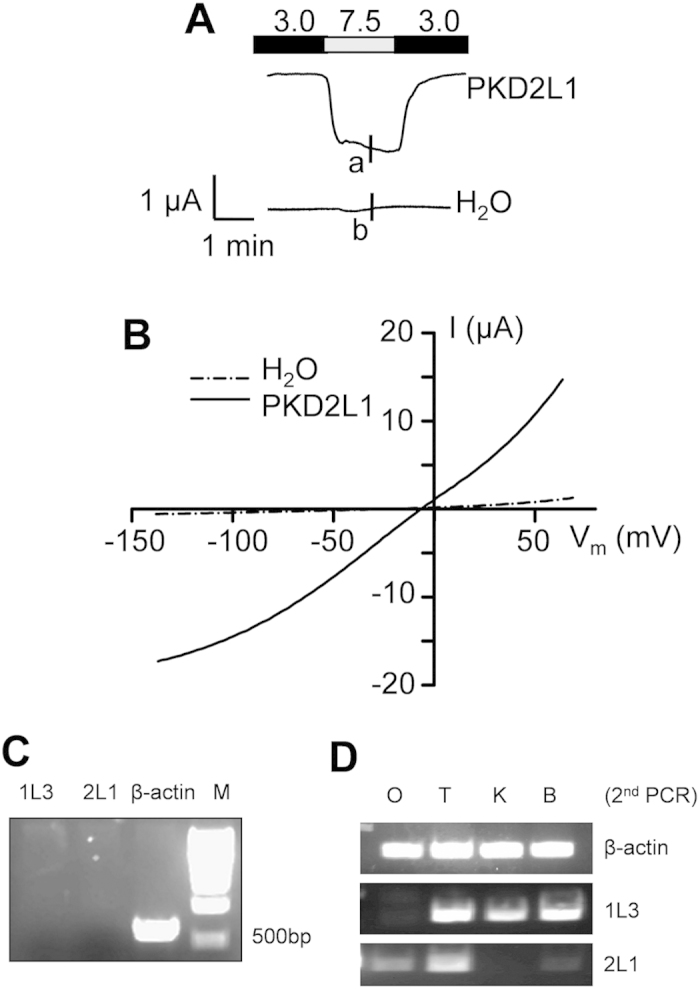
Acid-induced off-response currents in *Xenopus laevis* oocytes over-expressing human PKD2L1. **(A**) Representative currents recorded by the two-microelectrode voltage clamp at −50 mV from an oocyte expressing human PKD2L1 or a water-injected oocyte (as a control). An off-response current is defined as the difference between the current amplitudes at pH 7.5 and pH 3.0. The pH values of the standard extracellular solutions are indicated. (**B**) Representative current–voltage relationship curves obtained using a voltage ramp protocol at the time points indicated by ‘a’ and ‘b’ in the panel A tracings. (**C**) RT-PCR using *Xenopus* oocytes detecting the RNA signals of PKD2L1 (2L1), PKD1L3 (1L3) and β-actin. The predicted sizes are 658 bp, 706 bp and 718 bp for β-actin, PKD1L3 and PKD2L1, respectively. (**D**) Second (nested) PCR detecting PKD1L3 and PKD2L1 signals using *Xenopus* oocytes (O), tongue (T), kidney (K) and brain (B). β-actin signals served as positive controls.

Although reported acid-dependent off-response properties have so far all been associated with the co-expression of PKD2L1 and PKD1L3^15^,^18^,^23^,^24^,^25^,^26^,^27^,^28^, it has remained unclear as to the role of PKD2L1 in acid sensing and induction of the off-response. We employed the two-microelectrode voltage clamp electrophysiology to investigate how PKD2L1 expressed alone in *Xenopus* oocytes responds to acidic stimuli. In the absence of extracellular Ca^2+^ (to avoid the on-response effect of Ca^2+^ on PKD2L1), we found that increasing extracellular acidity (from pH 7.5 to pH 2.5–4.5) to PKD2L1-expressing or control oocytes does not induce appreciable currents. However, in PKD2L1-expressing, but not water-injected control, oocytes voltage clamped at −50 mV a large inward current was elicited following substitution of an acidic extracellular solution with a control solution at pH 7.5 (Fig. 1A). This large current induced in an off-response manner resembled those associated with PKD2L1/PKD1L3 co-expressed in HEK cells, oocytes and TRCs^15^,^16^,^18^,^25^. This indicates that PKD2L1 alone is able to exhibit off-response activation. We also voltage clamped oocytes at other membrane potentials using a voltage ramp protocol, which produced the acid-elicited off-response currents as function of the membrane potential (I-V curves) (Fig. 1B).

To examine whether any oocyte endogenous PKD1L3 contributed to our observed off-response currents we performed RT-PCR assays using total RNAs isolated from oocytes and various *Xenopus* tissues including the tongue, kidney and brain. No PKD1L3 or PKD2L1 signal was detected in oocytes in the first round of RT-PCR (Fig. 1C). We followed up with nested PCR assays and revealed a weak PKD2L1 signal in oocytes and brain, but still no detectable PKD1L3 signal in oocytes (Fig. 1C,D). Thus, the off-response observed in PKD2L1-expressing oocytes is independent of *Xenopus* PKD1L3.

**Figure 2:**
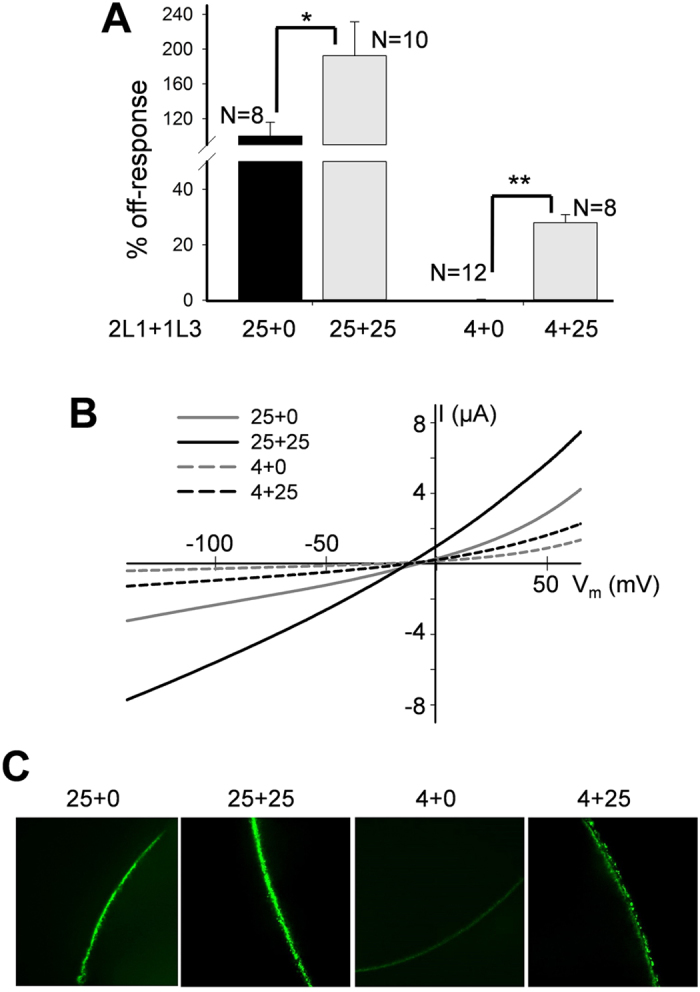
Off-response currents in oocytes expressing PKD2L1 with or without PKD1L3. (**A**) Averaged and normalized off-response currents elicited by extracellular pH 3.0 from oocytes injected with 4 or 25 ng of PKD2L1 (2L1) mRNA alone or co-injected with 25 ng of PKD1L3 (1L3) mRNA, and voltage clamped at −50 mV. Shown are off-response currents averaged from different numbers of oocyte, as indicated. *p = 0.05 and **p = 0.004, unpaired t-test. (**B**) Representative off-response I-V curves obtained using a ramp protocol under the same conditions as in panel A. (**C**) Representative immunofluorescence data showing the PM intensity of PKD2L1 protein in oocytes injected with different amounts of mRNAs, as indicated.

We next examined the effect of PKD1L3 co-expression on PKD2L1-induced off-responses. In oocytes injected with a small amount (4 ng per oocyte) of PKD2L1 mRNA the off-response was observed only in the presence of PKD1L3 co-expression (injected at 25 ng mRNA per oocyte) (Fig. 2A,B). Our immunofluorescence experiments showed that PKD1L3 co-expression increases the PM localization of PKD2L1 (Fig. 2C). These data are in agreement with a previous report in which similar amounts of mRNAs were injected^16^. In oocytes injected with a larger amount (25 ng per oocyte) of PKD2L1 mRNA, co-expression of PKD1L3 still increased off-response currents and PKD2L1 PM localization (Fig. 2). Thus, taken together, our data indicated that PKD2L1 exhibits the off-response when its PM localization exceeds a certain threshold level and that PKD1L3 enhances the off-response amplitude, likely through increasing the PM population of PKD2L1.

### Effects of acid dose, application time, and type on the PKD2L1 off-response

**Figure 3:**
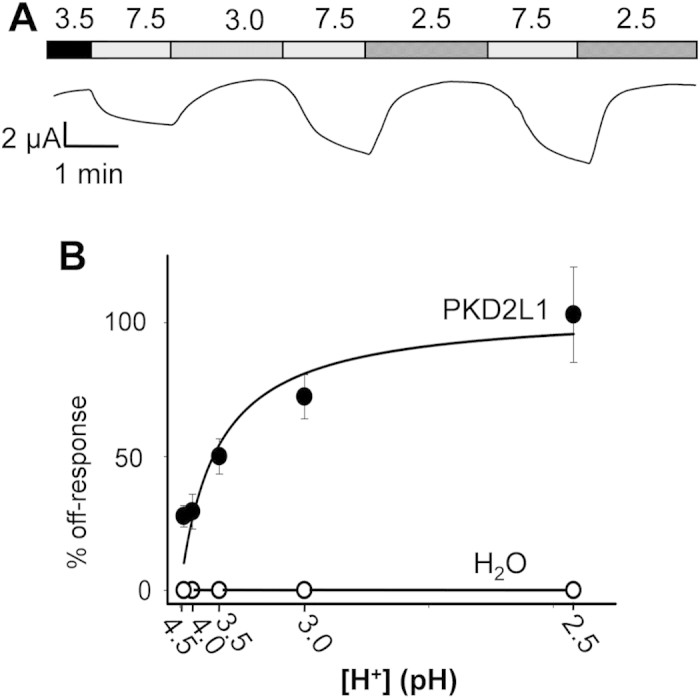
Dose dependence of the acid-induced off-response. (**A**) representative current recorded at −50 mV and different extracellular pH, as indicated, from a PKD2L1-expressing oocyte. (**B**) pH dose dependence of the off-response currents for oocytes injected with PKD2L1 mRNA or water. [H^+^] indicates protons concentration. Data points represent average values from N = 5–9 with the standard error of the mean (SEM) and were fitted to the Michaelis Menton Equation, which produced a K_m_ value of pH 3.5 ± 0.5.

Our voltage clamp data showed that the PKD2L1-associated off-response is dose-dependent with half maximal activation value of pH 3.5 ± 0.5 (N = 5–9) in PKD2L1-expressing oocytes voltage clamped at −50 mV (Fig. 3A,B). Previous studies also found dose-dependent off-responses, with cell-type dependent half maximal activation pH values. PKD2L1/PKD1L3 expressed in HEK cells responded to pH 3.0 or lower^15^,^18^,^24^, taste cells responded to solutions of pH 5.0 or lower^19^,^20^, while mouse CSF-cN neurons responded to changes in extracellular pH in the 6.5 to 7.4 range^20^.

**Figure 4:**
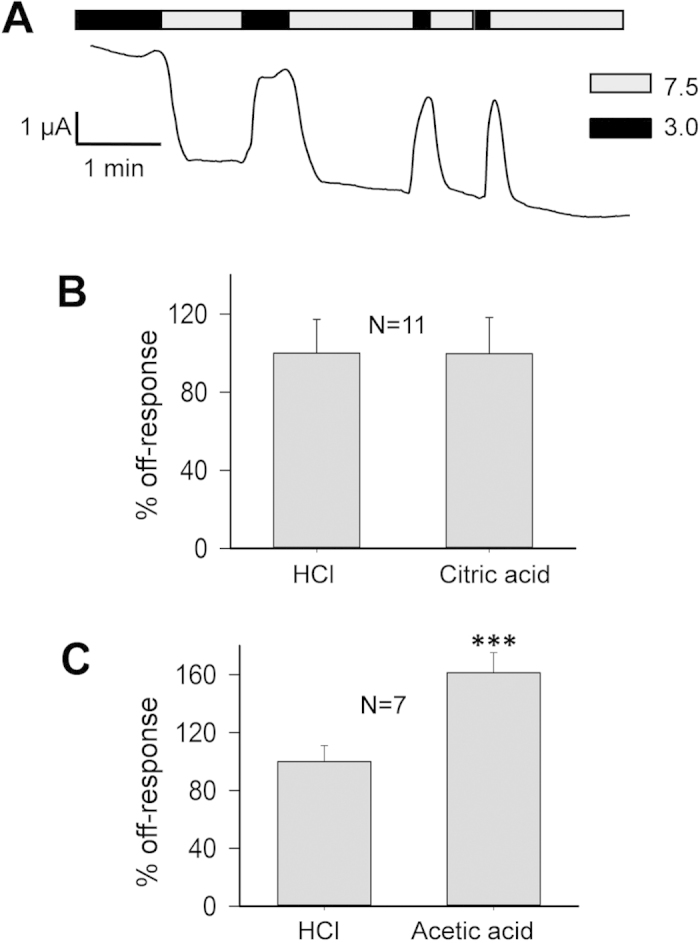
Effects of acid application time and type on the off-response. (**A**) Representative current recorded at −50 mV and with the application of the standard solution at pH 3.0 for different time intervals in an oocyte expressing PKD2L1. (**B**) Averaged and normalized off-response currents elicited by HCl or citric acid at pH 3.0 were compared from the same oocytes. (**C**) Averaged and normalized off-response currents elicited by HCl or acetic acid at pH 3.0 were compared from the same oocytes (***p = 0.0004, paired t-test).

In order to determine whether the off-response is sensitive to the duration of acid application, we applied a solution of pH 3.0 for different periods of time. We found that oocytes over-expressing PKD2L1 exhibit similar off-response amplitudes for different application durations, ranging from 10 sec to 1 min (Fig. 4A). Our data indicated that acid application time is rather not a determinant of the off-response amplitude, resembling the previously reported duration-independent characteristic of the PKD2L1/PKD1L3 channel complex^15^.

Further, we investigated whether there is any difference between weak and strong acids with respect to the amplitude of off-response current. For this we compared the effects of acetic and citric acids with HCl, using 50 mM Na-acetate or Na-citrate to replace 50 mM NaCl in the standard solution. These solutions were adjusted to pH 3.0 by titration with HCl. Considering the pK_a_ values of both acids, the resulting solutions should contain 49.1 and 12.9 mM of the undissociated membrane-permeable form of acetic and citric acids, respectively. Additionally, we found that the off-response current induced by acetic acid, but not citric acid, is significantly larger than that induced by HCl (60%, N = 7, p = 0.0004, paired t-test) (Fig. 4B,C). This pattern of increase is comparable to the response in the chorda tympani of rat TRCs by acetic, citric and hydrochloric acids at the same pH^29^.

### Roles of extracellular protons in modulating PKD2L1 on-response

**Figure 5:**
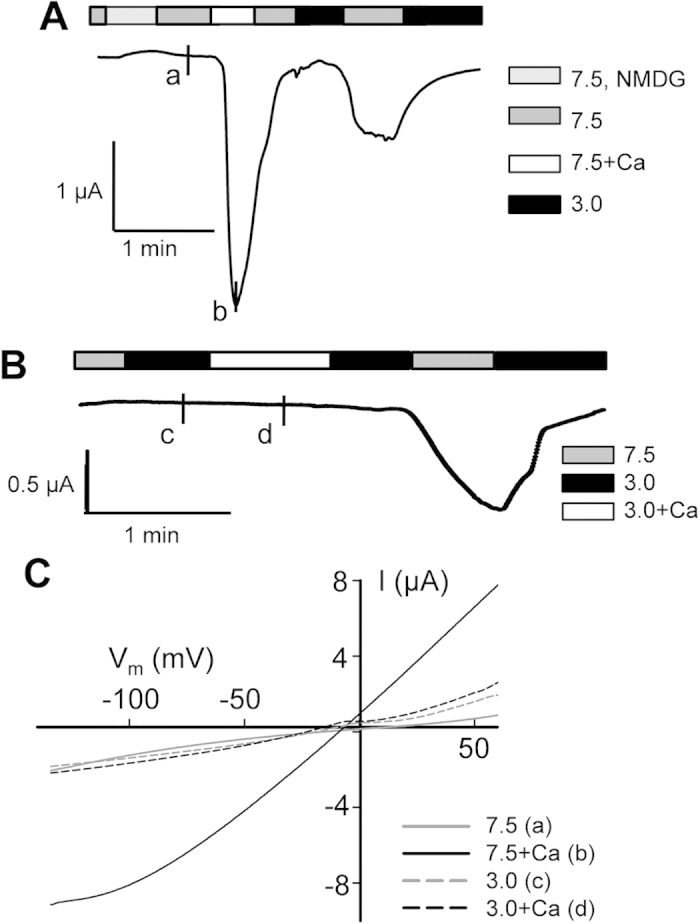
Acid-induced vs Ca^2+^-induced channel activation of PKD2L1. (**A**) Representative current recorded at −50 mV in a PKD2L1-expressing oocyte to show the on-response induced by 5 mM extracellular Ca^2+^ (at pH 7.5) and the subsequent off-response induced by extracellular pH 3.0 (no extracellular Ca^2+^). (**B**) Representative current recorded at −50 mV in a PKD2L1-expressing oocyte to show the on-response induced by 5 mM extracellular Ca^2+^ (at pH 3.0) and the subsequent off-response induced by extracellular pH 3.0 (no extracellular Ca^2+^). (**C**) I–V curves generated at the time points (‘a’–‘d’) indicated in panels **A and B**.

It was previously reported that application of extracellular Ca^2+^ induces PKD2L1 channel activation followed by an ensuing channel inactivation^5^. In contrast, an acid-induced off-response did not display an ensuing inactivation under Ca^2+^-free conditions (Fig. 5A). Thus, the same channel expressed in oocytes exhibits two apparently distinct properties: Ca^2+^-induced on-response with inactivation and acid-induced off-response (in the absence of Ca^2+^) without inactivation.

Extracellular low pH was previously found to significantly reduce Ca^2+^-induced PKD2L1 activation^5^. Consistently, here we found that the Ca^2+^-induced activation is completely abolished when extracellular pH drops to pH 3.0 (Fig. 5B). Similar conclusion can be made by comparing between the on-response current-voltage relationships obtained at pH 7.5 and 3.0 (Fig. 5C). Thus, the off-response activator, acid, played an inhibitory role in the on-response.

### Roles of Ca^2+^ ion in modulating PKD2L1 off-response

**Figure 6:**
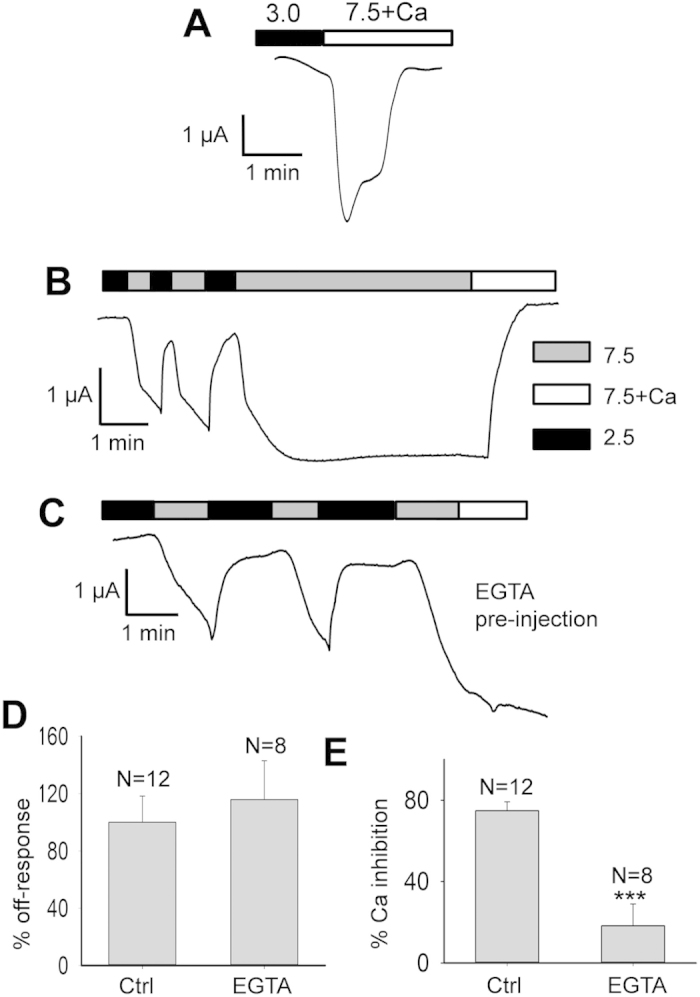
Roles of Ca^2+^ ions in the PKD2L1 off-response. (**A**) Representative tracing recorded at −50 mV, showing the off-response activation and ensuing inactivation in the presence of extracellular Ca^2+^ (5 mM). (**B**) Representative current recorded at −50 mV in a PKD2L1-expressing oocyte, showing the inhibition of the off-response current by extracellular Ca^2+^ (5 mM). (**C**) Representative current recorded at −50 mV in a PKD2L1-expressing oocyte 1 hr after injection of 50 nL of 50 mM EGTA, showing the effect of extracellular Ca^2+^ (5 mM). (**D**) Averaged and normalized off-response currents with EGTA or water (Ctrl) pre-injection. **(E**) Averaged percentage of the off-response current inhibition by extracellular Ca^2+^ with EGTA or water pre-injection. ***p = 0.0007, unpaired t-test.

Reversely, we wondered whether and how the on-response activator Ca^2+^ plays a role in the off-response. So far, we have performed all the off-response experiments in Ca^2+^-free extracellular environments, in part to avoid the Ca^2+^-induced on-response. Interestingly, in the presence of extracellular Ca^2+^ (5 mM), PKD2L1-expressing oocytes responded with a robust off-response current followed by channel inactivation, forming a spike of current (Fig. 6A) that has a similar shape as those previously published using HEK cells expressing PKD2L1/PKD1L3 in the presence of Ca^2+^ 15. Thus, compared with the presence of a sustained off-response current plateau in the absence of Ca^2+^ (see Figs 1A and 4A), our data together suggest that the off-response inactivation is attributed to Ca^2+^. Indeed, consistently, we found that sustained off-response currents were abolished by addition of Ca^2+^ (Fig. 6B). Thus, the on-response activator Ca^2+^ played an inhibitory role in the off-response.

Because of the permeability of PKD2L1 to Ca^2+^ we wondered whether the Ca^2+^-induced inhibition/inactivation of the off-response is due to an increase in the intracellular Ca^2+^ concentration ([Ca^2+^]_i_). For this, we pre-injected *Xenopus* oocytes over-expressing PKD2L1 with 50 nL of 50 mM ethylene glycol tetra-acetic acid (EGTA) tetra -sodium salt solution 1 hr before the same electrophysiological measurements as those for Fig. 6B, to chelate the intracellular Ca^2+^. We found that while EGTA pre-injection does not prevent PKD2L1 from exhibiting the off-response, it substantially reduces the ability of extracellular Ca^2+^ to inhibit the off-response (Fig. 6C–E). In average, EGTA pre-injection resulted in similar off-response currents compared with the control condition (115.8 ± 27.1%, N = 8–12, p = 0.6), while it reduced the Ca^2+^ inhibition ability to 23.1 ± 12.0% (N = 8–12, p = 0.0007, unpaired t-test) (Fig. 6D,E). These results suggest that the increase in [Ca^2+^]_i_ is critical for the extracellular Ca^2+^-induced inhibition, but not for the activation, of the off-response.

**Figure 7:**
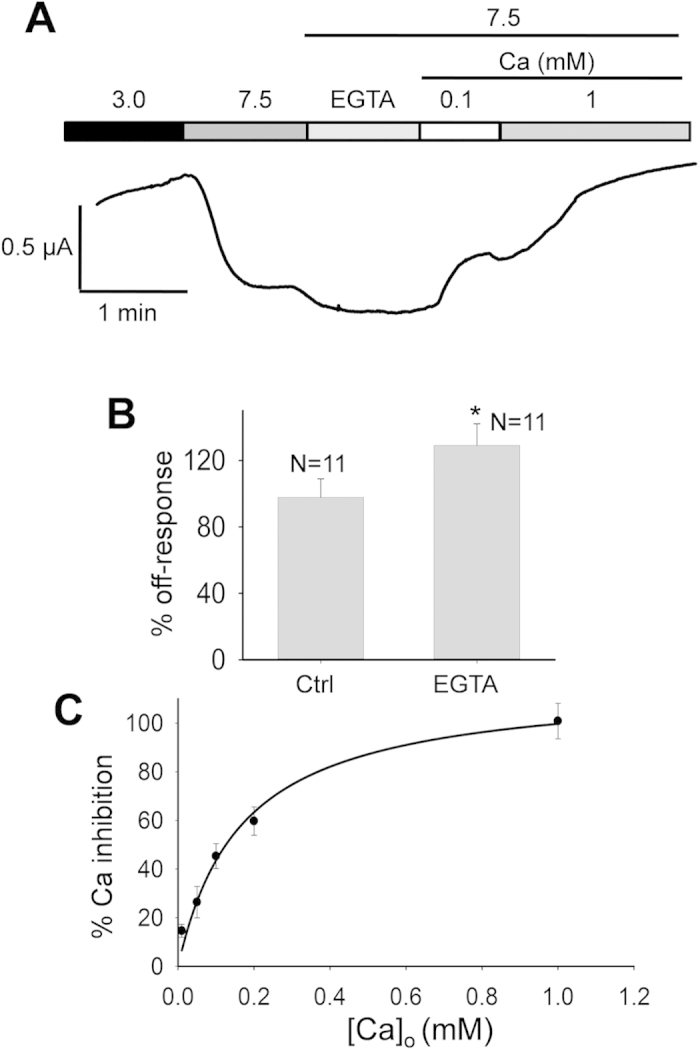
Role of extracellular Ca^2+^ ions in the PKD2L1 off-response. (**A**) Representative current recorded at −50 mV in a PKD2L1-expressing oocyte under various extracellular conditions, as indicated. (**B**) Based on experiments described in panel A, averaged data to show the effect of extracellular EGTA (1 mM). *p = 0.04, unpaired t-test. (**C)** Dose-dependence of the relative Ca^2+^ inhibition of the off-response current. Data were averaged from 11 oocytes and the curve is a fit of the data to Equation 1, with K_i_ value of 167 ± 33 μM.

We also used EGTA to chelate extracellular Ca^2+^. We added 1 mM EGTA to our extracellular solutions to chelate any trace amounts of Ca^2+^ ion and, consistently, found a 36.7 ± 3.8% (N = 11, p = 0.04, unpaired t-test) increase in the off-response current (Fig. 7A,B). We went further to determine the K_i_ value of the extracellular Ca^2+^ concentration ([Ca^2+^]_o_) for the Ca^2+^ inhibition by applying different [Ca^2+^]_o_ values, as shown in Fig. 7A. We found that 1 mM CaCl_2_ is sufficient to completely inhibit the off-response current and that K_i_ for [Ca^2+^]_o_ is equal to 167 ± 33 μM (N = 11) for the off-response current generated from pH 3.0 to 7.5 (Fig. 7C). This K_i_ value was obtained by fitting experimental data to the equation


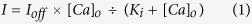


where *I*_*off*_ represents the off-response current and *I* is the inhibited fraction of *I*_*off*_. Of note, we think that the presence of trace amounts of ‘contaminating’ Ca^2+^ in extracellular solutions (~5 μM, based on data sheets of MgCl*2*, NaCl and KCl) may account, at least in part, for the difference in the off-response current with and without extracellular EGTA (Fig. 7A).

### Roles of acidic residues in PKD2L1 on- and off-responses

**Figure 8:**
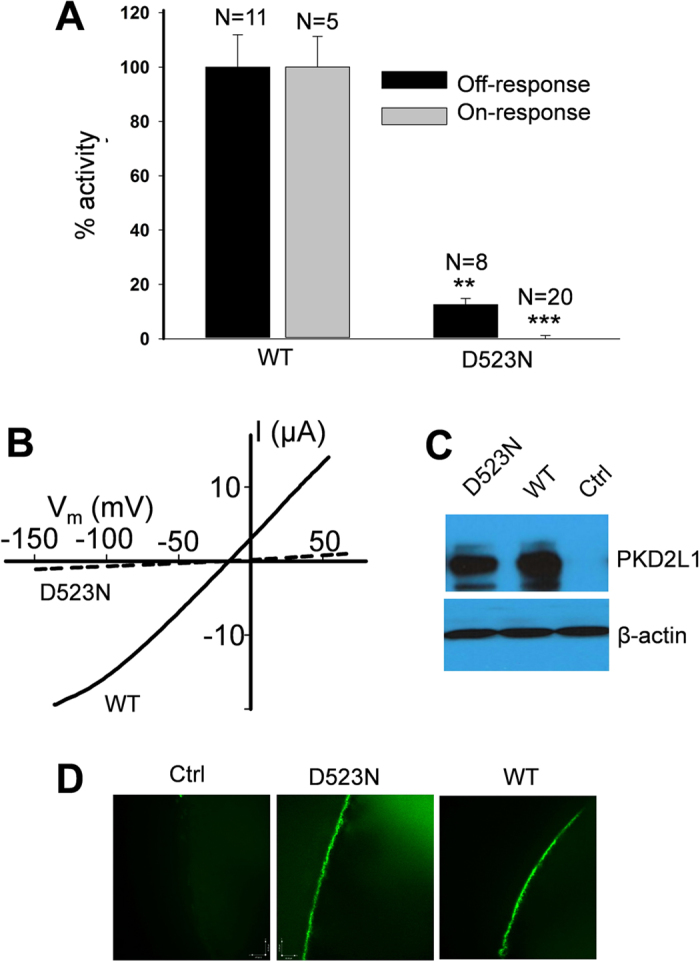
Effects of mutation D523N on the PKD2L1 on- and off-responses. (**A**) Averaged and normalized on- and off-response currents elicited by extracellular 5 mM Ca^2+^ and pH 3.0, respectively, from oocytes expressing WT or mutant PKD2L1 voltage clamped at −50 mV. Shown are on- (***p = 3.0e-6, unpaired t-test) and off-response currents (**p = 0.002, unpaired t-test) averaged from different numbers of oocyte, as indicated. (**B**) Representative off-response I-V curves obtained by a ramp protocol under the same condition as in panel A. (**C**) Western blotting data showing the protein expression of WT and mutant PKD2L1 in expressing or water-injected (Ctrl) oocytes. (**D**) Representative immunofluorescence data showing the PM localization of WT and mutant PKD2L1 in oocytes.

Previous reports found the importance of aspartic acid residue D523 in human PKD2L1 for the Ca^2+^ permeability^13^ as well as for the off-response of the PKD2L1/PKD1L3 complex^16^,^26^. We wanted to explore whether this mutation affects the Ca^2+^-induced on-response as well. We found that the D-to-N mutation inhibits the on- and off-response currents by 104.0 ± 1.6% (N = 20, p = 3.0e-6, unpaired t-test) and 77.2 ± 2.8% (N = 8, p = 0.002, unpaired t-test), respectively (Fig. 8A,B). We verified by Western blotting and immunofluorescence that the mutant is expressed on the PM to a similar extent as the wild type (WT) PKD2L1 (Fig. 8C,D). Thus, our studies found that residue D523 is essential for both on- and off-responses of PKD2L1.

**Figure 9:**
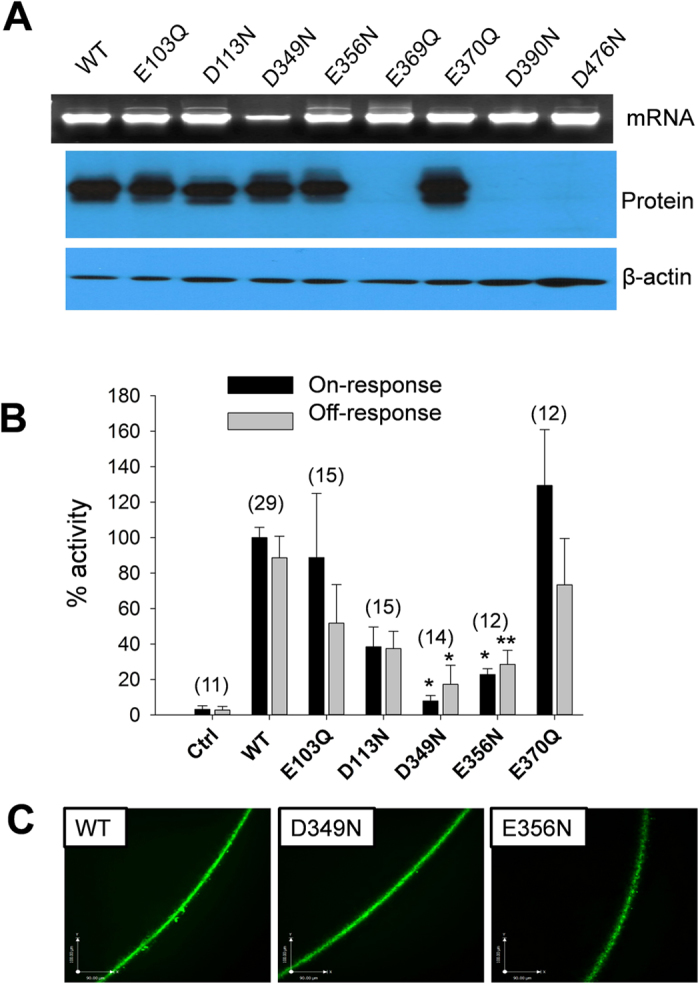
Effects of negatively charged intramembrane residues on the PKD2L1 on- and off-responses. (**A**) Representative data showing mRNA and protein bands that were revealed in 1% agarose gel and 8% SDS-PAGE, respectively. (**B**) Averaged and normalized on- and off-response currents elicited by extracellular 5 mM Ca^2+^ and pH 3.0, respectively. Oocytes expressing WT, mutant or water-injected oocytes (Ctrl) were voltage clamped at −50 mV. The number of each group is indicated in a bracket. On-response currents were significantly reduced, with *p = 0.03 and 0.05 (unpaired t-test) for D349N and E356N, respectively. Off-response currents were also significantly reduced, with *p = 0.05 and **p = 0.01 (unpaired t-test) for D349N and E356N, respectively. (**C**) Representative immunofluorescence data showing the PM localization of PKD2L1 WT, D349N and E356N expressed in oocytes.

We next examined the importance of intra-membrane acidic residues for PKD2L1 on- or off-response properties. For this we mutated eight such residues into their corresponding neutral residues: E103Q and D113N in TM1, D349N, E356N, E369Q and E370Q in TM3, D390N in TM4, and D476N in TM5, and then examined their expression and channel function in oocytes. We found that mutants E369Q, D390N and D476N have no detectable protein expression although their mRNA is normal (Fig. 9A), possibly rapidly degraded due to mis-folding. While the function of mutants E103Q, D113N and E370Q were similar to that of the WT channel, we found that the function of mutants D349N and E356N is significantly reduced (Fig. 9B). As our immunofluorescence data indicated that their PM levels are not affected (Fig. 9C), our electrophysiology data indicated that residues D349 and E356 in TM3 are critical for both the Ca^2+^-induced on-response and H^+^-induced off-response, possibly through interaction with Ca^2+^ or protons. Of note, the loss of function by point mutations D523N, D349N and E356N further supports the specificity of the off-response currents observed for WT PKD2L1 expressed alone in oocytes.

## Discussion

In the present study we have examined acid-dependent properties of human PKD2L1 expressed in *Xenopus* oocytes using the two-microelectrode voltage clamp electrophysiology, together with molecular biology and mutagenesis techniques. We found that PKD2L1 over-expressed alone in oocytes exhibits acid-induced off-response characteristics, such as pH dependence, activation by strong and weak acids, and I-V relationships, that are comparable to those previously reported for the PKD2L1/PKD1L3 complex in HEK cells and oocytes. Given the non-detectable PKD1L3 in oocytes, this indicates that PKD1L3 is not required for acid sensation of PKD2L1. PKD2L1 off-response was inhibited and regulated by an increase in intracellular Ca^2+^. Moreover, the intra-membrane residues D349 and E356 were found to be essential for channel function.

Previous reports showed that PKD1L3 is essential for the off-response through increasing the targeting of PKD2L1 to the PM^15^,^16^,^23^,^24^. Nonetheless, the functional roles of PKD1L3 in the channel activity of the PM PKD2L1/PKD1L3 channel complex remain unclear. It was proposed that the PKD1L3 long extracellular N-terminus is involved in sensing extracellular pH^15^. While PKD2L1 knockout mice have defective sour tasting, PKD1L3 knockout mice do not exhibit tasting abnormality^17^,^21^. This fact appears to be controversial and argues that PKD1L3 does not play a significant role in the PKD2L1/PKD1L3 complex in terms of sensing acid in the tongue. Studies by Yu and colleagues^16^, nicely supported our finding that PKD2L1 alone in oocytes is expressed on the PM. However, injection of ~4 ng mRNA of PKD2L1 into each oocyte did not result in measurable PKD2L1-associated currents in our study as well as in a previous report^16^. This is likely due to an insufficient PM population of PKD2L1. Co-expression of PKD1L3 much increased the PM level of PKD2L1, allowing PKD2L1 to exhibit measurable off-response whole-cell currents^16^. In fact, this report showed that tetrameric PKD2L1 is present on the PM even with a small mRNA amount injected and in the absence of PKD1L3. In addition, the same report indicated that PKD1L3 alone does not target to the PM or exhibit channel activity^16^. Therefore, these data together suggest that PKD2L1, rather than PKD1L3, may possess an acid sensing capability and forms an off-response channel. However, we could not exclude the possibility that an endogenous partner protein participates in the acid sensing in cells expressing PKD2L1 alone or complex PKD2L1/PKD1L3. In addition to the chaperone role, it is nevertheless possible that PKD1L3 modulates PKD2L1 channel properties. It is noted that another PKD1 homologue PKD1L1 indeed modulates the single-channel conductance of PKD2L1 in mammalian cells, though PKD2L1 alone exhibits channel function on the PM^13^.

PKD2L1 was first reported in 1999 to be a non-selective cation channel activated by and highly permeable to Ca^2+^ in *Xenopus* oocytes^5^, a property that we call here a Ca^2+^-induced on-response. Its Ca^2+^ permeability in mammalian cilium membrane is important for regulating ciliary Ca^2+^ concentration and Ca^2+^-dependent hedgehog signalling. However, distinct channel properties were observed in the primary cilium, such as outward rectification and absence of Ca^2+^-induced channel activation^13^,^14^. Whether the Ca^2+^-induced on-response and acid-induced off-response share part of the molecular mechanisms remains unknown. However, some different properties between the on- and off-responses are noted. For example, after an on-response a waiting/recovery period of 5–8 minute was required for the induction of a new on-response^5^. In contrast, as shown here, the off-responses (at least under Ca^2+^-free conditions) can be repeatedly induced immediately one after another. Further, Ca^2+^-induced channel activation was followed by channel inactivation while acid-induced channel activation was sustained without inactivation in the absence of Ca^2+^. These data also suggest that the Ca^2+^-dependent on-response and acid-dependent off-response are not completely governed by the same mechanism.

In fact, acid-induced off-response activation in PKD2L1-expressing oocytes occurred in the absence of extracellular Ca^2+^ and reached a plateau, similar to reported data using oocytes^16^. In fact, in the presence of extracellular Ca^2+^, the off-response was still present but was followed by an ensuing inactivation, producing a current spike that is similar to the reported observations in HEK cells for PKD2L1/PKD1L3 under a comparable condition^15^. Furthermore, if Ca^2+^ was added during an off-response activation, then an immediate inhibition of the off-response current was observed, with a K_i_ value (50% inhibition) for [Ca^2+^]_o_ of only 0.17 mM. These data together indicated that extracellular Ca^2+^ regulates (somehow inhibits) the off-response currents. In contrast, other previous reports using HEK cells expressing PKD2L1 and PKD1L3 stated that the acid-induced off-response requires the presence of extracellular Ca^2+^ and results in an increase in [Ca^2+^]_i_ 25. The discrepancy in the role of extracellular Ca^2+^ will have to be clarified in further studies.

Interestingly, while preloading oocytes with Ca^2+^ chelator did not significantly alter the off-response, it abolished the ability of extracellular Ca^2+^ to inhibit the off-response. This suggests that an increase in the intracellular free Ca^2+^ is required for Ca^2+^ to inhibit the off-response activation. On the other hand, we previously reported that an increase in intracellular Ca^2+^ is required for both the Ca^2+^-induced on-response activation and the ensuing inactivation^5^. In terms of the effect of intracellular protons, our data and previous report^29^ using weak and strong acids support that acid-induced off-responses positively correlate with increased intracellular H^+^ concentrations. Putting all data together, although Ca^2+^ and acid distinctly induce PKD2L1 channel activations they seem to share the same or similar way of inactivation, i.e., by an increase in [Ca^2+^]_i_.

It has remained unclear as to how Ca^2+^ and proton ions trigger channel activations although Ca^2+^ plays roles in both phenomena. It is worthy to note that the PKD2L1 C-terminus possesses an EF hand, a coiled-coil domain, a cytoplasmic regulatory domain (CRD, aa 561–805) and phosphorylation sites^30^,^31^. PKD2L1 CRD was shown to be related to channel inactivation^32^, maybe through direct binding to Ca^2+^, or through activating downstream signalling molecules. More recently, CRD binding to Ca^2+^ was reported to change the α-helical property of the C-terminus^30^, which may explain the desensitization/inactivation phenomenon that follows the Ca^2+^-induced activation. However, whether the same domain is involved in the Ca^2+^ inhibition associated with acid-dependent off-response needs further studies. On the other hand, we found two negatively charged residues D349 and E356 to be critical for both on- and off-response functions. Because aspartate and glutamate residues have their pK_a_ values of pH 3.7 and pH 4.0, respectively, which are well within the pH range in which PKD2L1 exhibits the off-response, our work suggests that protons may interact with these residues as part of the off-response activation process. Further, because of their importance for the Ca^2+^-induced on-response activation, these acidic residues may also interact with Ca^2+^ during the on-response process.

In summary, in the present study we discovered that PKD2L1 expressed alone in *Xenopus* oocytes exhibits extracellular acid-induced off-responses with characteristics resembling those associated with PKD2L1/PKD1L3 and that this off-response is inhibited by an increase in the intracellular Ca^2+^. Further studies will help better understanding how PKD1L3 and Ca^2+^ modulate the PKD2L1 channel function.

## Materials and Methods

### Preparation of *PKD2L1* plasmid constructs

Human PKD2L1 gene was cloned as previously described^6^. Mutations of aspartic and glutamic acid residues to the neutral residues asparagine or glutamine were performed using QuikChange Lightning site-directed mutagenesis kit (Agilent Technologies, La Jolla, CA, USA). Primer design and procedure were performed following the manufacturer’s protocol.

### mRNA preparation and micro-injection into *Xenopus laevis* oocytes

Plasmids pCHGF harbouring WT or a mutant *PKD2L1* cDNA were linearized with Mlu I, followed by phenol/chloroform purification and ethanol precipitation.

Linearized plasmids were used to *in vitro* synthesize capped mRNAs using the T7 mMessage mMachine^TM^ kit (Ambion, Austin, TX, USA). Stage V-VI oocytes were isolated from *Xenopus laevis* under an approved institutional protocol. Defolliculation of oocytes was performed through incubation in Barth’s solution^6^ containing Type 2 collagenase (2 mg/mL) (Worthington, Lakewood, NJ, USA) at room temperature for 1.5 hours (hr). Oocytes were then incubated at 16–18 °C in Barth’s solution supplemented with 1% antibiotics (penicillin/streptomycin) (GIBCO™, Life Technologies, Burlington, ON, Canada) for at least 3 hr before injection of 50 nL H_2_O containing 25–50 ng mRNAs or as stated in the experiment. An equal volume of H_2_O was injected into control oocytes. Injected oocytes were incubated at 16–18 °C in Barth’s solution, for 2–4 days prior to experiments. For intracellular Ca^2+^ chelation studies, oocytes were injected with 50 nL of 50 mM EGTA (Sigma-Aldrich, Oakville, ON, Canada) to allow a final intracellular concentration of ~5 mM 1 hr prior to recordings.

### Western blotting

Western blotting was performed as previously described^6^. Briefly, 10–20 μg of purified oocyte proteins were resolved on SDS PAGE (8%) gel, transferred to nitrocellulose membrane. Membrane was blocked with 3–5% skim milk in Tris Buffered Saline, 1% Tween 20 (TBST) for 1 hr at room temperature. This was followed by a 4 °C overnight incubation with rabbit anti-PKD2L1 polyclonal antibody (cat# PAB5914, Abnova, Taipei, Taiwan) diluted 1:1000 in the blocking buffer. Loading control β-actin was detected using a mouse monoclonal antibody (cat# sc-47778, Santa Cruz Biotechnology, Dallas, TX, USA). Secondary horseradish peroxidase (HRP) -coupled anti-mouse and anti-rabbit were purchased from GE Healthcare (Baie d’Urfe, QC, Canada).

### Immunofluorescence

*Xenopus* oocytes were washed in PBS, fixed in 3% paraformaldehyde for 15 min, and washed 3 times in PBS plus 50 mM NH_4_Cl, and then permeabilized with 0.1% Triton X-100 for 4 min. Oocytes were then washed 3 times in PBS for 5 min each time, blocked in 3% skim milk in PBS for 30 min, and then incubated overnight with the PKD2L1 polyclonal antibody. This was followed by 3 times 10 min washes in PBS. The oocytes were then incubated with a secondary AlexaFluor 488-conjugated donkey anti-rabbit antibody (Jackson ImmunoResearch Laboratories, West Grove, PA, USA) for 30 min, followed by 3 times washes in PBS and mounting in Vectashield (Vector labs, Burlington, ON, Canada). The slides were examined on an AIVI spinning disc confocal microscopy (Cell Imaging Facility, Faculty of Medicine and Dentistry, University of Alberta).

### Solution preparation

The standard extracellular solution contained (in mM): 100 NaCl, 2 KCl, 1 MgCl_2_, 10 HEPES adjusted to pH 7.5 by TRIS base. Low pH (2.5–4.5) standard solutions were prepared from the standard solution adjusted by HCl. Solutions containing extracellular Ca^2+^ were prepared from the standard solution with the addition of CaCl_2_.

### Electrophysiology

Current and voltage signals were measured with the conventional two-microelectrode voltage clamp technique with a commercial amplifier (TEV-200A, Dagan, Minneapolis, MN, USA). Electrodes were fabricated from borosilicate glass (Warner Instruments, Hamden, CT, USA) by a micropipette puller (P-87, Sutter Instruments, Novato, CA, USA) and filled with 3 M KCl with typical tip resistance of 0.5–3 MΩ. Digidata 1320A converter and pClamp 9.2 (Axon Instruments, Union City, CA, USA) were used for data acquisition and analysis. In experiments using a ramp or gapfree protocol^5^, current/voltage signals were digitized at 200 ms/sample. Recorded tracings were then analyzed using pClamp 9.2 and plotted using SigmaPlot 12 (Systat Software, San Jose, CA, USA). Absolute amplitudes of channel activity were collected and normalized to the average activity of WTPKD2L1 obtained under the same day, condition and group. Normalized values were then expressed as a percentage value and plotted using SigmaPlot 12.

### Reverse Transcriptase –Polymerase Chain Reaction (RT-PCR)

Total RNAs were prepared from female *Xenopus laevis* oocytes, tongue, kidney, and brain tissues. Oocytes were collected and defolliculated as described above. Tissues were washed in PBS and kept on ice. RNAs were then extracted at room temperature with TRIzol reagent (Invitrogen Life Technologies, Burlington, ON, Canada). According to the manufacturer’s manual, 1 mL TRIzol was used to homegenize 50–100 mg of tissue. Chloroform was used to isolate the RNA followed by precipitation, wash and resuspension steps. One-step RT-PCR was carried out using SuperScript^®^III One-step RT-PCR kit (Invitrogen Life Technologies) by following the instruction manual. The oligonucleotide primers for *Xenopus laevis β-actin* and *PKD1L3* for the first PCR were as follows: *β-actin*, sense GAGATGAAGCTCAAAGCAAAAG and antisense CAGGATTCCATACCAAGGAAAG; *PKD1L3* sense GCAGATTGTGAGGAAGAAAGG and antisense TGCTGAGAGCTGGTAGGGTAGT. 10X diluted first *PKD1L3* PCR products were used as templates to run the second (nested) PCR using inner primers to increase PCR specificity and efficiency. Sequences of the *PKD1L3* inner primers were: sense AGATTGTGAGGAAGAAAGGGGG and antisense TTTGCTAAAGTCTGGTGGGTTG.

### Statistical Analysis

Data were presented as mean ± (SEM). All data were collected from at least three different experiments. Student’s t-test was used to compare two groups of data for statistical significance indicated by a p value. P values of less than 0.05 and 0.01 were considered significant and very significant, respectively.

## Additional Information

**How to cite this article**: Hussein, S. *et al.* Acid-induced off-response of PKD2L1 channel in *Xenopus* oocytes and its regulation by Ca^2+^. *Sci. Rep.* **5**, 15752; doi: 10.1038/srep15752 (2015).
